# Two possible mechanisms of ganciclovir for treatment of major depressive disorder

**DOI:** 10.3389/fpsyt.2023.1109723

**Published:** 2023-04-25

**Authors:** Kazuhiro Itoh, Hiroshi Tsutani, Yasuhiko Mitsuke, Hiromichi Iwasaki

**Affiliations:** ^1^Department of Internal Medicine, National Hospital Organization Awara Hospital, Awara, Japan; ^2^Division of Infection Control and Prevention, University of Fukui Hospital, Fukui, Japan

**Keywords:** ganciclovir, major depressive disorder, human herpesvirus 6B, interferon beta, neuroinflammation, interferon stimulated genes

## 1. Introduction

The annual prevalence of major depressive disorder (MDD) in the US is 6.7% (1), and an estimated 35 million US adults will be affected by MMD during their lifetime (1–3). However, MDD is a global problem with an economic burden estimated at 83.1 billion USD in 2000 (2, 4). Furthermore, the prevalence of psychiatric disorders, including MDD, has increased during the COVID-19 pandemic (5). MDD is considered to be a multifactorial disorder caused by both environmental and genetic factors, but the mechanism underlying its pathogenesis is not fully understood (6). It is likely that there are multiple underlying mechanisms of pathogenesis (2, 6), given the existence of multiple patient subgroups with different characteristics. Current treatments for MDD, including pharmacotherapy, have not yet achieved satisfactory results (7–9). We hypothesize that ganciclovir may be a potential therapeutic candidate for MDD based on not only its antiviral action, but also its modulation of innate immune pathways in the brain that are activated in response to stress.

## 2. Mechanisms of disease for the various human herpesviruses/their relationship with depression

### 2.1. HHV as a risk for developing MDD

#### 2.1.1. Herpes simplex virus

Few studies have examined the association between depression and herpes simplex virus (HSV) infection and reactivation (10). In a study of US adults, HSV-1 was not associated with an increased risk of depression, but HSV-2 was associated with an increased risk of depression (11). In a study of Finnish adults, HSV-1 infection was not associated with new-onset depression (12). In studies of adolescents and adults, depressed patients are more likely to engage in risky sexual behaviors and consequently develop HSV-2 infection (13–15). Sexually transmitted diseases (STDs) are associated with depression, and depressive symptoms tend to be more severe in patients with STDs (14, 15). The odds ratio for depression was higher in HSV-2 patients (OR 2.1, 95% CI 1.5–2.9), and HSV-2 may be both a cause and a consequence of depression, including the fact that STDs are associated with depression (13). Persistent psychological stress is a risk for activation of HSV-2 (16).

#### 2.1.2. Varicella-zoster virus

A cross-sectional cohort study of 104 older adults aged 60 years and older found that indices of varicella-zoster virus (VZV) cell-mediated immunity were significantly lower in a group of subjects with MDD than in an age- and sex-matched control group without a history of depression or psychiatric disorders (17). In a matched case-control study of subjects aged 50 years and older, 389 herpes zoster (HZ) cases and 511 matched controls were enrolled, and the adjusted odds ratio for depression was 3.81 higher in the HZ group than in the control group, and stress was a risk factor for HZ (aOR 2.80) (18). In a study conducted in Taiwan of HZ patients aged 18 years and older, 1,888 HZ patients were compared with 7,552 age- and sex-matched controls, and HZ patients were significantly more likely to develop MDD, and HZ was an independent risk factor for MDD (hazard ratio 1.49, 95% confidence interval 1.04–2.13) (19). Postherpetic neuralgia is associated with the development of depression (20).

#### 2.1.3. Epstein-Barr virus

In the study of Finns over 30 years of age cited above, Epstein-Barr virus (EBV) seropositivity was not associated with the risk of developing depression (12). On the other hand, several studies have suggested an association with depression. In a study of 87 patients with MDD and 312 controls, in which antibodies to EBV were measured by solid phase immunoassay and Western blotting, low levels of antibodies to EBNA-1 and high levels of antibodies to EBV virions increased the likelihood that the individuals would be diagnosed with MDD (21). These findings suggest that altered immunity to EBV may be associated with the immunopathology of MDD (21). In adolescent females, increased depressive symptoms are significantly associated with salivary shedding of EBV DNA (22). In studies of pregnant women, EBV reactivation has been associated with increased rates of depression (23, 24). Infectious mononucleosis is most commonly caused by EBV infection (25), but a study in Denmark found that infectious mononucleosis was associated with a higher risk of depression compared with unaffected individuals (HR 1.40, 95% CI 1.26–1.56) (26).

#### 2.1.4. Cytomegalovirus

In a study of 137 older adults in the United Kingdom, among the cytomegalovirus (CMV) seropositive group, the higher the CMV IgG, the more likely they were to be anxious and depressed (27). In a study in US adults, higher CMV antibody levels were associated with depression in CMV antibody-positive individuals (11). A study analyzing data from older US Latinos aged 60 years and older found that CMV seropositivity was significantly associated with an increased likelihood of developing depression (OR 1.38, 95% CI 1.00–1.90) (28).

#### 2.1.5. Human herpesvirus 6

Human herpesvirus 6 (HHV-6) is reactivated from latent infection in the cerebellum of patients with MDD (29). HHV-6B is widespread around the world, including in Europe, the US, and Japan, with primary infection occurring between the ages of 6–12 months, followed by latency in the human body (30). This latent HHV-6B infection has been reported to produce a small latent protein, encoded by the intermediate stage transcript of HHV-6-1 (SITH-1), in olfactory bulb astrocytes. SITH-1 forms a complex with calcium-regulated cyclophilin ligand to cause calcium influx into the cell and induce apoptosis. In SITH-1 model mice, SITH-1 is produced by olfactory bulb sheath cells, a type of olfactory astrocyte, leading to apoptosis in the olfactory bulb and the expression of depressive symptoms. Patients with MDD have been found to show significantly higher detection of SITH-1-specific antibodies compared with healthy controls, with an odds ratio of 12.2 (31). Overexertion leads to increased HHV-6B in saliva, which can increase the number of SITH-1-producing cells (32). Among patients with MDD, late proteins indicative of HHV-6B activity and viral DNA have been detected in the cerebellum 80 and 53% more frequently than in controls, respectively (29).

### 2.2. HHV as a risk for worsening MDD

In a study of antibody titers to HSV, CMV, and EBV in 65 patients with coronary artery disease, the greater the severity of depression, the higher the rate of seropositivity to latent viruses (33). Cytomegalovirus infection is associated with decreased volume of gray matter in the brain in patients with MDD. This result suggests that cytomegalovirus infection may be a treatable cause of structural brain abnormalities in depressed patients (34). SITH-1-induced olfactory bulb apoptosis may also facilitate HHV-6B or other HHVs invasion into the brain, which is associated with worsening depressive symptoms (31). In a study of 11- to 18-year-olds in Turkey, depressed patients with suicidal ideation had significantly higher levels of HHV-6 antibodies, suggesting that persistent HHV-6 infection may be a risk factor for suicidal ideation (35).

The adult prevalence of HHV in the general population is that HSV-1 infects about 70% of adults, HSV-2 about 30%, VZV more than 90%, EBV also more than 90%, CMV about 70%, and more than 95% of those 2 years and older are infected with either HHV-6A or HHV-6B or both (36). In contrast, in the depressed population, a study examining serologic testing in Turkish adolescents found HSV-1 in 71.4%, EBV in 82.9%, CMV in 94.2%, and HHV6 in 91.4%, and no statistically significant difference in healthy controls in the same study (35). No significant differences in HHV morbidity are expected between the general population and the depressed population, and it is likely that some infected individuals are more susceptible to depression than others. Therefore, HHV infection alone cannot be considered a risk factor for depression.

## 3. Antiviral mechanism of intervention and previous studies on efficacy for the various viruses

Although HHV-6 has no established treatment, anti-cytomegalovirus agents including ganciclovir are known to be effective (37, 38). Ganciclovir is also effective against herpesviruses such as HHV-1, herpes zoster virus, and Epstein–Barr virus (38–40). In an open label study of valganciclovir (a prodrug of ganciclovir), 75% of patients with high immunoglobulin G antibody titers to HHV-6 and Epstein–Barr virus and four or more of the following symptoms for at least 1 year, cognitive dysfunction, slow processing speed, sleep disturbance, short-term memory impairment, fatigue, and symptoms consistent with depression, achieved almost complete resolution of symptoms, and all returned to work or full-time activities (41). A phase I clinical trial of valganciclovir treatment for CMV positive patients with MDD is currently underway (ClinicalTrials.gov Identifier: NCT04724447). Based on these findings, it is conceivable that the antiviral effect of ganciclovir on herpesviruses is beneficial in the treatment of MDD.

## 4. Effects of ganciclovir on neuroinflammation with STING pathway activity

The relationship between stress and MDD is well-known, with stress causing activation of the brain's innate immune response pathways (42). Stimulator of interferon genes (STING), an adaptor protein expressed in microglia, plays an important role in regulating innate immune signaling processes in the central nervous system by detecting abnormal cytoplasmic DNA (43). Cyclic guanosine monophosphate (GMP)-adenosine monophosphate (AMP) synthase (cGAS) catalyzes the generation of 2′3′-cyclic-GMP-AMP (cGAMP), a second messenger that binds and activates STING. STING then recruits and activates TANK-binding kinase 1 and the transcription factor interferon regulatory factor 3 to produce interferon-β (IFN-β) (43). In an experimental mouse model of chronic restraint stress, decreased levels of STING and activation of its downstream molecules were observed in the hippocampus and prefrontal cortex (44). In addition, the mice exhibited depression-like behavior and elevated levels of the inflammatory cytokines tumor necrosis factor α, interleukin (IL)-6, and IL-1β in the brain (44). Activation of STING by the agonist cGAMP was shown to enhance phagocytosis of microglia in the brains of the mice, suppress the release of inflammatory cytokines, and exert antidepressant effects (44). Ganciclovir inhibited neuroinflammation by stimulating INF-β production in microglia depending on the STING pathway activation level (45). These findings suggest that the second point of action of ganciclovir is to promote phagocytosis of microglia by increasing INF-β production through activation of the STING pathway, which may lead to the improvement of MDD symptoms by suppressing neuroinflammation.

## 5. FDA data on ganciclovir adverse events

FDA data have reported depressive symptoms were observed in 27 (0.59%) of the 4,593 people, especially 40–49-year-old women, treated with ganciclovir from 1997 to 2022 (46). However, many of these patients were infected with cytomegalovirus, human immunodeficiency virus, or had acute lymphocytic leukemia (hematological malignancy), and many were also steroid users (46). It is possible that these patient characteristics were highly associated with depressive symptoms, and the mechanism of the association with ganciclovir is not clarified. Therefore, whether ganciclovir treatment causes depression requires careful interpretation. Side effects other than depression reported in the FDA data included cytomegalovirus infection, febrile neutropenia, pancreatitis, stress and anxiety, decreased hemoglobin, decreased weight, decreased hematocrit, thrombocytopenia, nosebleed, and urinary tract infection (46). Ganciclovir is primarily indicated for the treatment of CMV, so its administration to depressed patients is not indicated (47). In addition, it is administered with caution to patients with psychiatric disorders, and informed consent should be obtained prior to use in a clinical trial. Drug label information does not specifically list interactions with antidepressants, but warnings generally list hematologic toxicity, reproductive impairment, fetotoxicity, mutagenicity, and carcinogenicity (47).

## 6. Limitations

Neuroinflammation alone is not enough to explain MDD; psychological, social, environmental and cultural factors are also involved (48–51). Several clinical trials of anticytokine therapy for the neuroinflammatory hypothesis have been reported. Two clinical trials of the TNF-α inhibitor infliximab in depression showed no overall significant efficacy (52, 53). In addition, a meta-analysis of anticytokine therapy found a significant antidepressant effect, but the subjects were patients with chronic inflammatory diseases such as psoriasis and Crohn's disease, and depressive symptoms were measured only as a secondary outcome, so the results cannot be generalized as a therapeutic effect of anticytokine therapy for depressed patients (54). Toll-like receptor (TLR) 2/4 has been shown to be an important mediator of microglial activation in the medial prefrontal cortex by repeated social defeat stress, leading to neuronal and behavioral changes through inflammatory cytokines (55). The antimicrobial agent minocycline has been shown to inhibit inflammatory cytokine production by blocking phosphorylation of downstream molecules in the TLR 4 pathway (56). A pilot study of adjunctive minocycline treatment in patients with treatment-resistant depression showed an improvement in depressive symptoms in the minocycline group compared to placebo (57), but a randomized controlled trial of the efficacy of minocycline and celecoxib in combination or as monotherapy in bipolar depression found no significant difference in either group compared to placebo (58). Thus, at this time, clinical trials have not demonstrated sufficient power to support the neuroinflammation hypothesis. In addition, as noted above, HHV infection does not explain all causes of MDD.

## 7. Conclusion

**Figure 1:**
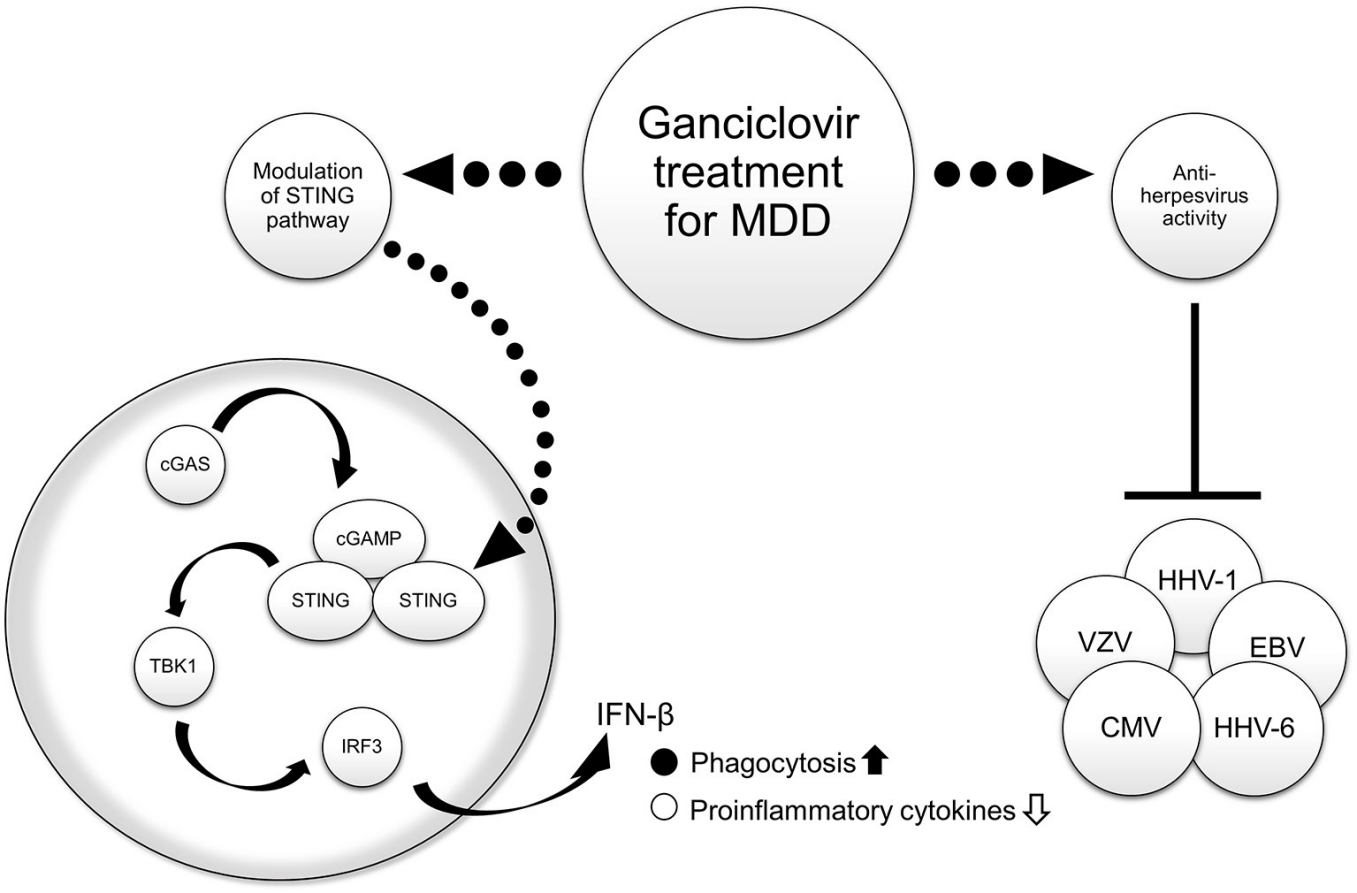
Hypothesized mechanisms of the therapeutic effect of ganciclovir in major depressive disorder (MDD). The right side of the figure shows antiviral activity, and the left side shows that activation of the STING pathway in central nervous system cells leads to the suppression of neuroinflammation by promoting phagocytosis and inhibiting inflammatory cytokine production. Source: Figure made by the authors with reference to Poole et al. (38) and Duan et al. (44). cGAS, cyclic guanosine monophosphate (GMP)-adenosine monophosphate (AMP) synthase; cGAMP, 2′3′-cyclic-GMP-AMP; CMV, cytomegalovirus; EBV, Epstein–Barr virus; HHV, human herpesvirus; IFN-β, interferon beta; IRF3, interferon regulatory factor 3; MDD, major depressive disorder; STING, stimulator of interferon genes; TBK1, TANK-binding kinase 1; VZV, varicella-zoster virus.

We reviewed data from basic (*in vitro* and *in vivo*) and observational studies on MDD and HHV, as well as basic neuroinflammation experiments (*in vitro* and *in vivo*) on MDD and the STING pathway. In addition, we presented an early-stage study on the potential therapeutic use of ganciclovir for MDD. Ganciclovir may be a potential therapeutic candidate for MDD from two different perspectives: antiviral activity against herpesviruses and inhibition of neuroinflammation through activation of the STING pathway, as shown in Figure 1. If we can measure the presence or absence of viral infection and antibody titers or the degree of neuroinflammation in patients with MDD as future work, we may be able to select subjects with MDD for whom ganciclovir is effective. We hope that validation by further clinical research can expand the range of treatment options because many patients with MDD still show a poor response to treatment.

## Author contributions

KI, HT, YM, and HI contributed to conception and methodology of the manuscript and wrote sections of the manuscript. KI performed data curation. KI and HI wrote the first draft of the manuscript. All authors contributed to manuscript revision, read, and approved the submitted version.
